# Non-rectangular neurostimulation waveforms elicit varied sensation quality and perceptive fields on the hand

**DOI:** 10.1038/s41598-023-28594-0

**Published:** 2023-01-28

**Authors:** Riccardo Collu, Eric J. Earley, Massimo Barbaro, Max Ortiz-Catalan

**Affiliations:** 1 Department of Electrical and Electronic Engineering, University of Cagliari, Cagliari, Italy; 2 Center for Bionics and Pain Research, Mölndal, Sweden; 3 Department of Electrical Engineering, Chalmers University of Technology, Gothenburg, Sweden; 4 Operational Area 3, Sahlgrenska University Hospital, Mölndal, Sweden

**Keywords:** Biomedical engineering, Excitability

## Abstract

Electrical stimulation of the nerves is known to elicit distinct sensations perceived in distal parts of the body. The stimulation is typically modulated in current with charge-balanced rectangular shapes that, although easily generated by stimulators available on the market, are not able to cover the entire range of somatosensory experiences from daily life. In this regard, we have investigated the effect of electrical neurostimulation with four non-rectangular waveforms in an experiment involving 11 healthy able-bodied subjects. Weiss curves were estimated and rheobase and chronaxie values were obtained showing increases in stimulation time required to elicit sensations for some waveforms. The localization of the sensations reported in the hand also appeared to differ between waveforms, although the total area did not vary significantly. Finally, the possibility of distinguishing different charge- and amplitude-matched stimuli was demonstrated through a two-alternative-forced-choice (2AFC) match-to-sample task, showing the ability of participants to successfully distinguish between waveforms with similar electrical characteristics but different shapes and charge transfer rates. This study provides evidence that, by using different waveforms to stimulate nerves, it is possible to affect not only the required charge to elicit sensations but also the sensation quality and its localization.

## Introduction

Human skin is a complex organ made up of different receptors that respond with different dynamics to interactions with the outside world^1–4^. Although each receptor is activated by stresses of a different nature, the natural sensation of touch is the result of a synergistic activation of the various receptors in the skin^5^. For individuals with nerve damage, this synergistic activation has been difficult to replicate artificially and is a topic of great research interest.

The restoration of sensory feedback is mainly based on the electrical stimulation of nerves using invasive or non-invasive techniques^6–9^. Invasive techniques are based on the stimulation of nerves using implantable electrodes such as spiral cuffs^10^, Flat Interface Nerve Electrodes (FINEs)^11,12^, Transverse Intrafascicular Multichannel Electrodes (TIMEs)^13^ or Utah Slanted Electrode Arrays (USEAs)^14^, or using epidural stimulation^15^. On the other hand, non-invasive techniques are based on the use of superficial electrodes positioned on the residual limb at the position of the main peripheral nerves (TENS)^16–18^. Whatever the kind of interface, electrical stimulation is typically performed using rectangular charge-balanced waveforms^19^. Rectangular waveforms are easily generated using the stimulators available on the market, even if they typically elicit sensations described as electrical or unnatural^20–23^. However, several studies have shown that modifying the geometrical parameters of the rectangular waveform can affect neuron excitability^24–26^ as well as modify the quality and location of sensations^27–29^.

The study of non-rectangular waveforms is less thorough than that of rectangular waveforms, possibly due to the more complicated implementation with the current technology. In fact, the main studies on non-rectangular waveforms are mainly related to computational models and computer simulations. In 1992, Wessale et al. performed a study comparing between rectangular and exponential waveforms, evaluating the strength duration curve of the two shapes^30^. Results showed that rectangular and exponential waveform exhibit differences in rheobase and chronaxie, and in particular that the rectangular waveform required a lower current to reach the threshold. This effect was explained by the phenomena of accommodation that entails an increase of threshold by using stimuli with slowly rising slopes. Wongsarnpigoon et al. evaluated six different waveforms in terms of delivered charge, energy, and power using computational models and an in-vivo experiment on a cat sciatic nerve^31^. This experiment showed that no one waveform can be efficient at the same time in all three selected parameters. Sahin and Tie developed a model to compare the physiological effect of non-rectangular waveforms with respect to the rectangular waveform, showing that the chronaxie of rectangular waveform is lower than those of non-rectangular waveforms^32^. Wongsarnpigoon and Grill developed a genetic algorithm to determine an energy-optimal shape that was identified in a truncated gaussian^33^. Foutz and McIntyre evaluated the effect of non-rectangular waveforms on deep brain stimulation using a computational model^34^. They identified the optimal stimuli in the centered triangular, gaussian and sinusoidal waveforms. However, while these previous studies investigated the physiological and psychophysical performance of these waveform shapes, no study has yet investigated the changes in sensation quality arising from the use of these waveform shapes.

In this study, we build upon these previous studies and examine the effect of non-invasive electrical stimulation with non-rectangular waveforms on a group of 11 able-bodied subjects. We developed three different tests analyzing the strength duration curve of 4 non-rectangular stimuli and the effect on the induced perception on the hand in terms of dimension and quality, defined as the subjective descriptor of what the sensation “feels like” irrespective of other aspects such as intensity or location. We show that significant differences in psychophysical response to stimulation can be observed depending on the stimulation waveform. Finally, we investigated the ability to distinguish sensations induced by stimuli with different waveforms, showing that the shape of stimuli can influence the kind of sensation reported on the hand.

## Methods

### Hardware

The waveform profiles were generated using MATLAB and transferred using the Standard Commands for Programmable Instruments (SCPI) to a function generator (33511B, Keysight, CA, USA). The use of the SCPI protocol was exploited to control the function generator from the computer, thus allowing the operator to fix the number of pulses of stimulation cycles, the duration of the stimulation, the type of waveform, and its characteristics in terms of shape, amplitude, and duration. The interface also allowed the user to collect information during stimulation, allowing the user to perform psychophysical tasks such as the two-alternative forced choice task.

**Figure 1:**
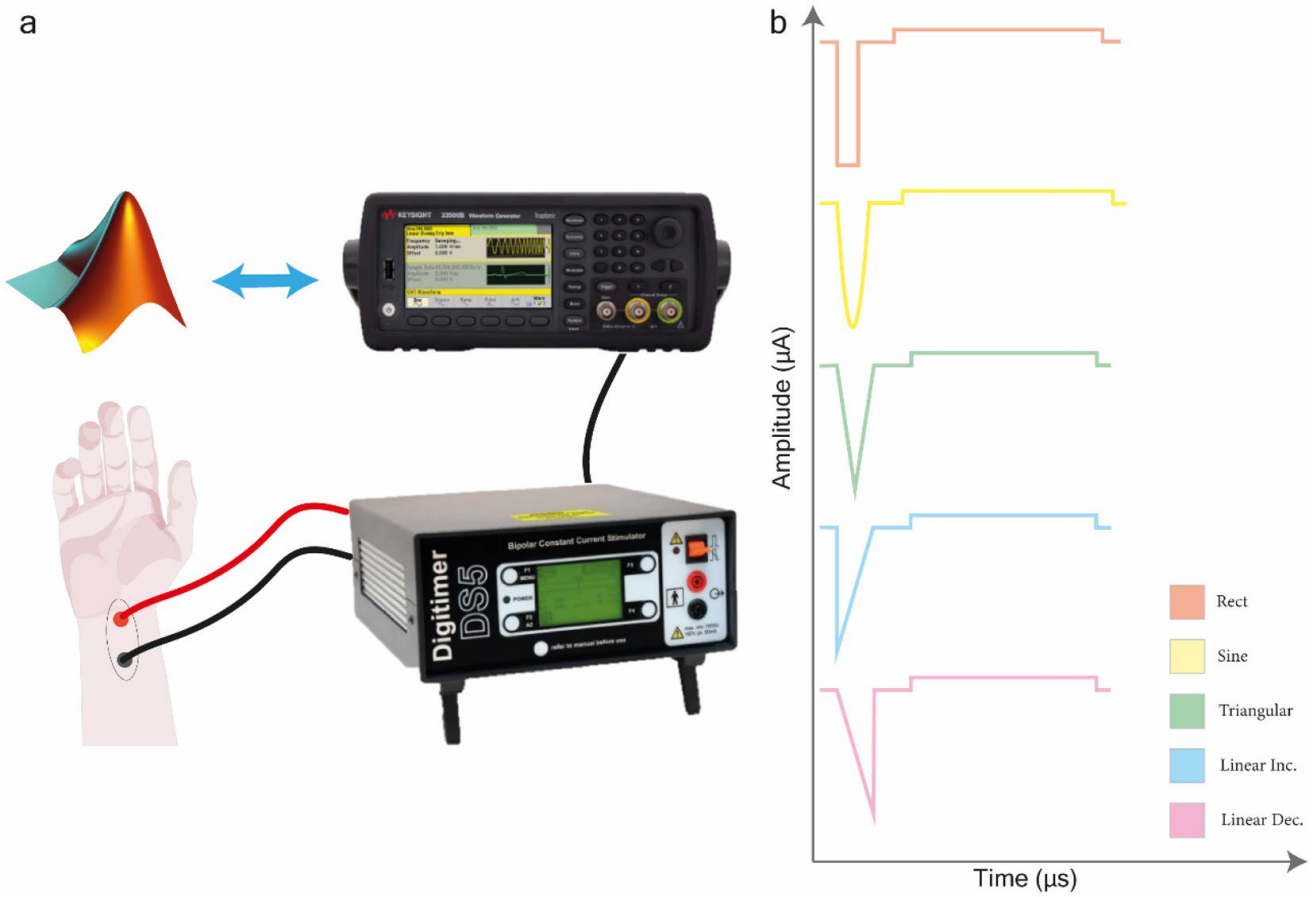
(**a**) The experimental setup is based on a MATLAB interface allowing the experimenter to control the Keysight function generator via serial connection and SCPI standard. The function generator is therefore used to generate the desired voltage waveforms and bring them to the input of the DS5 current stimulator, which is connected directly to electrodes placed on the subject's wrist directly over the median nerve. (**b**) The five waveforms tested in this study are rectangular, sinusoidal, triangular, linear increasing ramp, and linear descending ramp. The waveforms were charge-balanced with an interphase delay of 100us between the cathode phase and the anode phase.

Transcutaneous Electrical Nerve Stimulation (TENS) was delivered using an isolated bipolar constant current stimulator (DS5, Digitimer, England, UK). The output of the waveform generator was set as input for the DS5 providing current consistent with the desired waveform (Fig. 1a). DS5 calibration was performed as recommended by the manufacturer. The waveform generator was connected to DS5 input, while a 1kΩ resistor was connected to the current generator outputs. The generated waveforms were observed connecting an oscilloscope to the monitor output of DS5 and observing the corrected generation of anodic and cathodic phases for all the desired waveforms.

### Waveform shapes

The waveforms were generated to have biphasic waves with balanced charge and an inter-phase delay of 100 μs, which is considered safe for long-term neurostimulation^35^. Biphasic waves combined a variable waveform (rectangular or non-rectangular) cathodic phase and a rectangular anodic phase, where anodic amplitude and duration were selected to balance the total charge. Five different wave shapes were selected: rectangular (Rect), sinusoidal (Sine), triangular centered (TR), linear increasing ramp (LineInc) and linear decreasing ramp (LineDec), as shown in Fig. 1b Through the MATLAB interface, waveforms were generated by setting at least two parameters including charge, amplitude and duration of the cathodic stimulation phase.

The sine wave shape was modeled using the formula:

$$I(t)={A}_{cathodic}sin(t\frac{\pi }{PW})$$

where ${A}_{cathodic}$ is the peak current and $PW$ is the duration of the cathodic phase. The total charge of the cathodic phase was computed as:

$$Q={\int }_{0}^{PW}{A}_{cathodic}\mathit{sin}\left(t\frac{\pi }{PW}\right)dt=2{A}_{cathodic} \frac{PW}{\pi }$$

The area of the triangular shape and the two linear ramps was computed as:

$$Q={A}_{cathodic}\frac{PW}{2}$$

To compensate the charge of the cathodic phase, the anodic phase was generated fixing the amplitude to 10% of the cathodic amplitude. In this way, the duration of the anodic phase was computed as:

$$P{W}_{anodic}=\frac{10Q}{{A}_{cathodic}}$$

### Subjects and ethical approval

Eleven healthy subjects (6 females and 5 males) with an average age of 26 ± 3 took part in the trial. The number of required subjects was determined via a power analysis with effect size of 1.2, α = 0.05 and minimum power of 0.80. All subjects agreed to participate in the study and signed informed consent. The experimental protocol was approved by the Swedish regional ethical committee in Gothenburg (Dnr: 2019–05,446) and the research was performed in accordance with the relevant guidelines and regulations in compliance with the Declaration of Helsinki.

### Experimental protocol

The experimentation was carried out in time slots of 3 h. Participants were asked to sit with their arms placed on a table used as a support to maintain the position during the experiment. The stimulation electrodes were positioned 1.5 cm from each other at the median nerve on the wrist. Subjects were able to take a break at any time during the experiment, and at the end of every task a small break was done until the subject was ready to restart. During the entire procedure, the DS5 was periodically disconnected and calibrated using the auto-zeroing function to avoid possible dangerous DC currents.

### Detection thresholds

The experimentation was carried out in three different steps. In the first, we sought to determine the minimum current amplitude to elicit a sensation with the 5 different waveforms. Thresholds were obtained using a 1 up / 2 down adaptive psychophysics method with 50μA steps and a threshold set at 10 reversals. The threshold was obtained for 5 different stimulation durations (100 μs, 300 μs, 400 μs, 600 μs and 900 μs). This test was performed for stimulation with a single pulse and stimulation with a train of 15 pulses at 30 Hz.

Data obtained from the detection threshold was used to fit the Lapique’s equation, a relation describing the strength duration curve in other words, the relation between the minimum current required for stimulation and the pulse duration^36,37^. Lapique’s equation was fitted using robust linear least-square fitting method based on the bisquare weights method.

The effective amplitude (rms) of current was considered during the fit to make a reasonable comparison between the charge injected during stimulation and to apply the definition of Lapique’s equation:

$$I=b(1+\frac{c}{d})$$

where $b$ is the rheobase, $c$ is the chronaxie, and $d$ is the duration of cathodic phase. Detection thresholds were fitted to Lapique’s equation to estimate rheobase and chronaxie. The effective amplitudes of the non-rectangular waveform shapes were treated as an equivalent peak amplitude for the rectangular waveform; in this regard it was possible to apply the definition of the Weiss Eq. ^38^ to estimate the required charge to stimulate nerves and induce a recognizable sensation:

$$Q=b\left(d+c\right)$$

### Description of elicited percept

Once the thresholds of the different waveforms were obtained, participants described the sensations aroused in the hand, each with a cathodic phase duration of 400 μs, for both single pulses and trains of pulses. The 400 μs duration of the cathodic phase was selected to investigate a point in the middle of the range considered during the detection of the threshold. Moreover, using 400 μs duration guarantees the possibility to stay within safe stimulation limits^35^. The charge of the different waveforms was set to the charge of the rectangular waveform increased by 20%. During this phase, subjects received stimulations with the waveform under investigation and were subsequently asked to describe the location of the sensation in the hand and the sensation quality reported. All sensations reported by subjects were collected using a custom MATLAB interface. During the task subjects were able to ask for new stimuli until they were ready to describe what they perceived.

To better understand the variation in sensation area, the dimensions of sensations elicited by non-rectangular waveforms are compared to those elicited from rectangular waveform:

$$\Delta {A}_{\%}=\frac{100{(A}_{nonRec}-{A}_{Rec})}{{A}_{Rec}}$$

where ${A}_{nonRec}$ is the area of sensation elicited by the non-rectangular waveform and ${A}_{Rec}$ is the area of sensation elicited by the rectangular waveform.

### Two-alternative forced-choice match-to-sample

The third protocol asked participants to correctly distinguish between pairs of waveforms with different shapes. To ensure that the delivered charge and peak current were identical for all conditions, this protocol was only conducted with the triangular, linear increase, and linear decrease waveforms. Thus, any ability to distinguish between waveforms would be due entirely to the temporal profile of the waveforms. First, two stimuli were provided with different waveform shapes, with 5 s in between. Another 5 s after the second stimulus, a third stimulus was provided which matched the first or second. Subjects were asked to identify which stimuli were matched. This protocol was carried out using the same delivered charge in the second protocol. A sequence of 30 different combinations of stimuli were presented, thus allowing to have 10 samples for each pair of stimuli (TR-LineInc; TR-LineDec; LineInc-LineDec). The data from this two-alternative-forced-choice (2AFC) match-to-sample task were collected and the values were analyzed by means of an average of the responses reported by the subjects.

### Statistical analysis

To understand consistent dissimilarities between rectangular waveform and non-rectangular waveforms, we performed a null hypothesis test using Wilcoxon signed-rank test considering alpha significance level α = 0.05. For rheobase and chronaxie, where non-rectangular waveforms were compared to the rectangular waveform, four comparisons were performed, and Holm-Bonferroni corrections were applied. The Wilcoxon signed-rank test was also performed to determine if any significant variation in sensation area was induced by changing the stimulation waveform. For the third protocol, the one sample Wilcoxon signed-rank test determined if the discrimination success rate was greater than 50%.

## Results

### Rheobase and chronaxie

**Figure 2:**
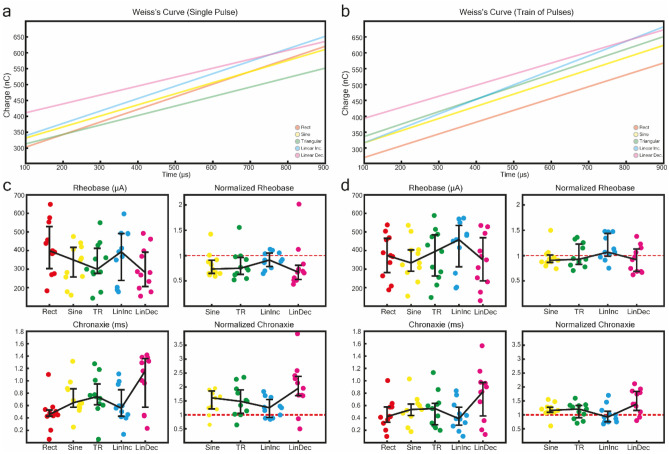
(**a**) Weiss’s curve fitting the data obtained from stimulation with single pulse. (**b**) Weiss’s curve fitting the data obtained from stimulation with train of 15 pulses at 30 Hz. (**c**) Rheobase and Chronaxie estimates from fitting Weiss’s curve to stimulation data with single pulse, displayed as both raw estimates (left two plots) and normalized with respect to Rect (right two plots). (**d**) Rheobase and Chronaxie estimates from fitting Weiss’s curve to stimulation data with train of 15 pulses at 30 Hz pulse, displayed as both raw estimates (left two plots) and normalized with respect to Rect (right two plots).

The data obtained in the detection threshold task were used to estimate the rheobase and chronaxie for the corresponding waveforms by means of Weiss’s equation (Fig. 2a,b). To obtain a valid comparison between the waveforms, strength-duration curve behavior is described in terms of effective current amplitudes (rms) instead of peak current amplitude.

To compare subject sensitivity to stimulation between waveforms, the modeled rheobase and chronaxie of non-rectangular waveforms were normalized with respect to the rectangular waveform as reported in Fig. 2c,d. Normalized values are reported in Table 1.

**Table 1:** Median and quartiles for normalized Rheobase current and Chronaxie time, with respect to Rect. Statistically significant differences are indicated in bold.

Shape	Single Pulse		Train of Pulses	
	Rheobase	Chronaxie	Rheobase	Chronaxie
Sine	0.73 [0.64,0.90]	**1.61 [1.21,1.85]**	0.9 [0.86,1.01]	1.15 [1.09,1.27]
TR	0.75 [0.62,0.96]	**1.48 [1.21,1.85]**	0.92 [0.82,1.22]	1.21 [0.89,1.33]
LinInc	0.91 [0.78,1.05]	1.25 [1.21,1.85]	1.07 [0.98,1.43]	0.91 [0.74,1.12]
LinDec	0.67 [0.52,0.8]	**1.95 [1.21,1.85]**	0.91 [0.68,1.13]	**1.38 [1.15,1.84]**

Differences in rheobase and chronaxie were quantified using a one-sample comparison with respect to the baseline (e.g., Rect). The normalized chronaxie for single pulses were significantly higher than Rect for LinDec (p = 0.0196), Sine (p = 0.0274), and TR (p = 0.0411), but not for LinInc (p = 0.0537). For trains of pulses, the normalized chronaxie was found significantly higher than Rect for LinDec (p = 0.027) but not for the other non-rectangular waveforms (p ≥ 0.09). No significant differences were found for the normalized rheobase during either single pulses (p ≥ 0.212) or during trains of pulses (p ≥ 0.4).

### Perceptive fields and sensation quality

**Figure 3:**
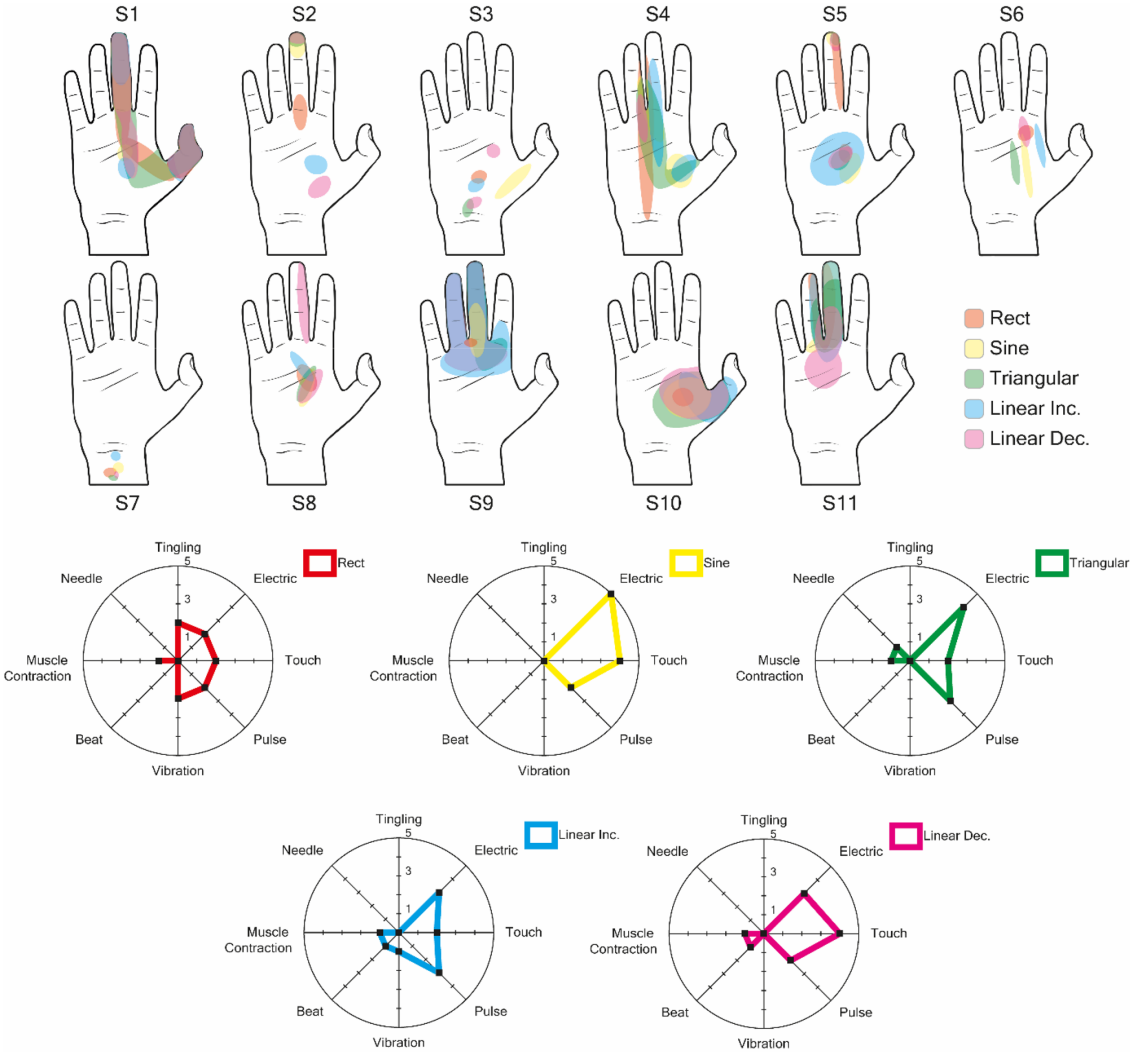
The sensations reported following single-pulse stimulation were reported and superimposed on the same drawing. Although in some cases the sensations share the same point of origin, it can be seen that as the type of waveform varies, the sensation area expands or moves, a phenomenon that could indicate a different activated neural portion or a greater number of fibers recruited. In addition, the sensations reported by the eleven volunteers were collected and graphed with respect to the type of waveform, showing how each waveform has different trends in terms of the type of sensation. Radar plots have been graphed inside the range of the sensation with the maximum correspondences. In particular, it appears that waveforms with greater peak amplitude were more likely to arouse electrical sensations.

**Figure 4:**
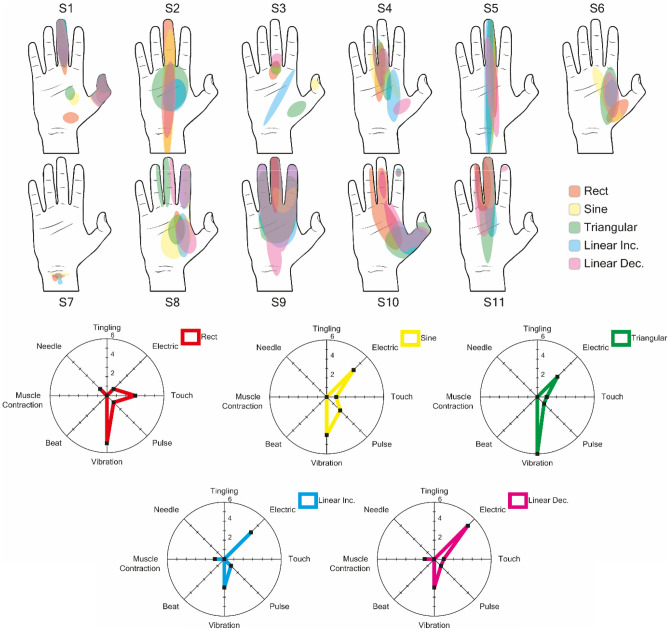
The sensations reported following stimulation by train of 15 pulses at 30 Hz were reported and superimposed on the same drawing. As in the case of single-pulse stimulation, it can be seen that the different waveforms have similar points of origin, although the areas of sensations are slightly different, and tend to be larger for non-rectangular waveforms. The increased delivered charge induced by the pulse train also results in sensations in a greater portion of the hand affected by the stimulation, indicating that more neural fibers have been affected. Radar plots have been graphed inside the range of the sensation with the maximum correspondences. In this case, moreover, the induced sensations are moving towards vibrational sensations, though for linear ramps the electrical sensations are still more predominant.

The percept localization task aimed to determine if different stimulations could recruit different neural fibers thus affecting the location and quality of the sensations. In Figs. 3, 4, the location of the sensations and the different qualities of sensations reported by the subjects with respect to the waveform used for stimulation are shown. The number of stimuli required by subjects to identify the quality and location of sensation was not recorded, although we note that subjects typically required only one cycle of stimulation to describe it. Non-rectangular waveforms reported a higher incidence of sensations perceived as electrical, a fact that may be linked to the higher peak current level and not to the charge injected during stimulation. No significant differences were reported between the dimensions of the elicited sensation from non-rectangular waveforms for single pulse stimulation (p ≥ 0.6) or train of pulses stimulation (p ≥ 0.096) with respect to rectangular waveform. Normalized sensation areas with respect to Rect induced areas are reported in Table 2.

**Table 2:** Median and quartiles for normalized change in sensation area.

Shape	Normalized Single Pulse Area	Normalized Train of Pulses Area
Sine	+ 1% [−22%, 85%]	+ 15% [1%, 115%]
TR	−4% [−38%, 43%]	+ 77% [9%, 104%]
LinInc	−9% [−23%, 134%]	+ 25% [−12%, 137%]
LinDec	−1% [−28%, 210%]	+ 27% [5%, 171%]

Generally, stimulation with trains of pulses resulted in a larger sensation area in the hand when compared to single pulses: Rect increased by 53% [−3%,353%], Sine 92% [8%,391%], TR 177% [−9%,630%], LinInc 141% [1%,310%], LinDec 130% [51%,323%]. This may be linked to the higher charge injected because of the higher number of pulses, even if the charge of the threshold was lower. Overall, these results suggest that while the dimension of elicited sensations may be more strongly related to the total injected charge, the sensation quality may be influenced by the shape.

### Two-alternative forced-choice match-to-sample

**Figure 5:**
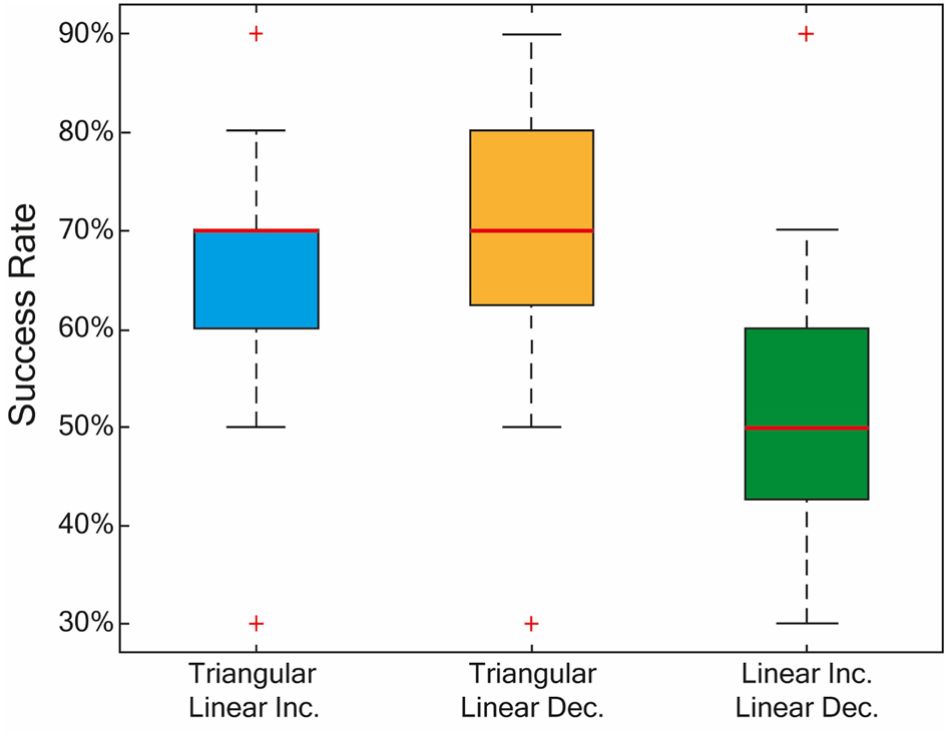
The data obtained by the 2AFC match-to-sample task were collected and analyzed. The results indicate that in about 70% of cases, volunteers were able to correctly distinguish the stimulation carried out with a linear or triangular ramp, indicating that the dynamics of the waveform can influence the induced sensation. On the other hand, it was more difficult for volunteers to distinguish between waveforms with similar dynamics as in the case of the comparison between linear ramps, in which the success rate was only 50%.

The 2AFC match-to-sample task was performed to comprehend whether the sensations induced by waveforms having the same charge and same peak current, but different dynamics were differentiable. To measure the capability to correctly distinguish between the waveforms the success rate of each comparison was compared to a baseline of 50% (i.e., random selection) using one-sided Wilcoxon’s signed-rank test. Significant increases in discrimination rate were found between Triangular and Linear Inc. (median success rate = 70%, p = 0.048) and Triangular and Linear Dec. (median success rate = 70%, p = 0.0176), while this was not observed between Linear Inc. and Linear Dec. (median success rate = 50%, p = 0.54). The data suggest it is generally possible to distinguish between triangular waveforms and either of the linear ramps (Fig. 5). Also interesting is the description given by the subjects who claimed to have concentrated on variations of “rhythm”, “intensity”, and “dimension” of the sensation to understand the difference, despite identical stimulation frequencies, amplitude, and duration of stimuli. The ability to discern between the two linear ramps, which present equal but opposite current slopes, was on average about 50% of success suggesting that they were indistinguishable. Overall, these results suggest that modulating stimulation shape could be another parameter by which to communicate information to an individual, in addition to modulating the pulse amplitude, pulse width, or stimulation frequency.

## Discussions

In this study, we investigated the effect of non-rectangular waveforms in non-invasive electrical nerve stimulation. To do this, we developed three different experiments (detection threshold, perception localization, and 2AFC discrimination) to answer three specific questions:

1. Does the shape of stimuli affect the physiological parameters that describe neuron excitability to electrical stimuli?
2. Does the shape of stimuli affect the kind of perception in terms of sensation quality, location, and dimension?
3. Is it possible to distinguish between the induced sensations of two stimuli with the same delivered charge, amplitude, frequency, and duration, but different shapes?

We found from the detection threshold task that the waveform shape has an influence on chronaxie but little influence on rheobase. This effect seems quieted by train of pulses stimulation, where rheobase and chronaxie are shifted closer to the values of rectangular waveform. From the perception localization task, we found that the shape can affect the sensation quality on the hand, and finally, from the 2AFC match-to-sample task we found that it is possible to discriminate between sensations elicited with different waveforms but same amplitude, frequency, charge, and duration. The results suggest that it is possible to influence the excitability of neurons and the capability to recruit different portions of nerve fibers by changing the shape of the stimulation waveform.

### Rheobase and chronaxie

We observed that for single pulse stimulation, the chronaxie for non-rectangular waveforms were generally higher than that of the rectangular waveform, and the rheobase for non-rectangular waveforms were generally lower than that of the rectangular waveform, although only the former trend was found to be statistically significant. For both rheobase and chronaxie, differences between non-rectangular and rectangular waveforms were smaller when using a train of pulses.

The results obtained from the detection of threshold, especially for single pulse stimulation, seem to be coherent with results shown by Sahin and Tie^32^, who showed how the waveform used during stimulation can have an effect on the stimulation efficiency and therefore influence neuron excitability, especially for chronaxie as shown in Fig. 2c. Similar to the findings obtained by Wessale et al., and Sahin and Tie, non-rectangular stimuli seemed to result in a higher chronaxie^30,32^.

The rheobase and chronaxie estimates were highly variable between subjects, which is a frequent issue especially for the chronaxie^39^. Knowing the values of rheobase and chronaxie of a subject with respect to stimulation shapes may help to develop more efficient and preferable stimulation algorithms, capable to stimulate nerves and induce a desired sensation while minimizing the charge required from the hardware and prolonging the battery life of the neurostimulation system.

### Perceptive field and sensation quality

Some interesting aspects emerge from the descriptions of elicited sensations, suggesting that the waveform may influence the induced sensation. The injected charge is not the only factor influencing neural recruitment, but stimulus dynamics and amplitude are also important. Lowering the peak amplitude appears to be a key factor in arousing sensations perceived as more natural and less electric. In fact, for single pulses, the number of subjects that described sensations as "electric" varied between 3/11 and 4/11 for non-rectangular waveforms, compared to 2/11 for the rectangular shape. With trains of pulses, rectangular waveforms had an incidence of electric sensations of 1/11 while for non-rectangular waveforms it ranged from 2/11 to 4/11. Even if non-rectangular waveforms appear to have a higher likelihood of eliciting electrical sensations, the variability of reported sensations suggests this is not guaranteed, thus changing the waveform may be an option to change the perceived sensations during stimulation. The obtained results suggest that not only the charge and duration of electrical stimuli, but also the shape of the waveform can affect sensation quality.

### Differentiation of waveforms

Interesting considerations emerge from the 2AFC task. Subjects were generally able to correctly identify between the triangular waveform and the linear ramps, which, although sharing identical delivered charge and peak and effective current amplitudes, showed different dynamics. The test also showed that participants were unable to differentiate between sensations elicited by the two linear ramp waveforms. This situation may be caused by the maximum rate of current change. In fact, the centered triangular has faster rates of current change than the two ramps, which featured equal but opposite current change rates. The capability to distinguish between different stimuli represent a big opportunity in neurostimulation modulation, since the possibility of using waveform shape as a new parameter to encode information may give a new degree of freedom in developing algorithms for sensory feedback.

### Limitations

Tests were conducted in 3-h slots for each subject within the same experimental session. This may have affected the subjects' ability to concentrate, even with frequent breaks, thus affecting the rheobase and chronaxie estimates and introducing a general noise in the threshold estimation This may have especially been the case regarding train of pulses which were always conducted after the single pulse study, leading to a general subject fatigue with a negative impact on concentration.

The large number of thresholds obtained (5 thresholds for 5 waveforms for 2 sessions) in a restricted time could also have led to adaptation effects to electrical stimulation^40^, thus increasing the charge necessary to feel a sensation in the waveforms of the final phase of experimentation. This may have minimized the differences of rheobase and chronaxie between non-rectangular and shapes. To obtain a greater detail of chronaxie and rheobase it may be necessary to perform detection threshold for single pulse and train of pulses in two different sessions and increase the number of thresholds measured for each task.

The data obtained during the description of elicited sensations should be verified more thoroughly through subsequent studies to verify the stability of the sensations in terms of quality and location during repeated and randomized stimulations. Randomizing the trials, it would be possible to minimize the possible error and bias introduced doing the stimulation with train of pulses always after single pulse stimulation. In this regard, it would be necessary to reproduce the characterization of sensation in a follow-up study. Collecting data over time, it would be possible to statistically investigate the effect of non-rectangular waveforms on elicited sensations with respect to rectangular waveforms. Moreover, this would lead a more detailed correlation between the amplitude of stimuli and the elicitation of electrical sensations.

It is also important to investigate how the results obtained during description of elicited sensations and the results of 2AFC depend on the positioning of the electrodes on the wrist and if results are reproducible in different types of stimulation, such as through the use of invasive electrodes. The reproducibility of the reported experiment using different kinds of interfaces would open the possibility to introduce a new degree of freedom in stimulation, allowing to use the variable waveform shapes during closed-loop control of bionic prostheses.

### Future directions

Although the results of this study are promising there are several aspects that should be investigated further. It would be interesting to evaluate the impact of electrode positioning on the arm to understand if these kinds of results can be obtained from positioning the electrodes in different locations and if it is possible to exploit this approach to maximize the effect of stimulation in situations in which the position of the electrode is determined by the prosthetic socket. Then, when the effect of electrode positioning has been evaluated, focus can be moved to people with amputations. The possibility to execute the experimental protocol with people with limb loss can also naturally be extended to direct nerve stimulation with invasive interfaces^41,42^.

The variability encountered in sensation localization could also be investigated in a larger sample size study to identify possible clusters in sensations distribution. Such a study should be accompanied by an investigation on the propagation of electrical signals generated by the different waveform in the nerve using finite element methods to associate the projection in the hand to the portion of nerve stimulated^43,44^.

The possibility of integrating stimuli with different waveform shapes into more complex algorithms such as biomimetic^45^ and neuromorphic^16^ stimulation should be investigated to understand if it is possible to improve the quality of stimulation and elicit more natural sensations. The combination of rectangular and non-rectangular stimuli should be considered in a follow up study to understand the trade-offs of mitigation of paresthetic and electric sensations. The combination of different stimuli should be investigated also to understand if multi-shape stimuli may lead not only on a modification of sensations but also impact on rheobase and chronaxie. Moreover, the implementation of linear amplitude modulation and frequency modulation for sensory feedback should be investigated, and the experiments proposed by Tan et al.^12^ and Ortiz-Catalan et al.^27^ should be reproduced to understand if the execution of patterned stimulation with non-rectangular waveforms has a different effect on quality and location of perceptions.

Another important topic that should be investigated is the impact of electromyographic control. The control of bionic hands is mainly based on recording and elaboration of electromyographic (EMG) signals from residual muscles of amputees^46–49^. The use of electrical stimulation can introduce electrical artifacts in EMG recordings leading to a failure in prosthetic control^50^. The impact of waveform shape on EMG signal should be investigated to understand if it is possible to minimize the effect of electrical artifacts induced by the stimulation process, as well as how they may interact with artifact removal algorithms used to denoise such a signal^51^.

## Conclusions

The data collected during this work suggest that non-rectangular waveforms can be viable alternatives to the classical rectangular biphasic shape. With respect to rectangular waveforms, using non-rectangular waveforms small variations in rheobase and chronaxie and different capabilities to stimulate nerves have been observed during this work. The discrimination between triangular shapes suggests the possibility to distinguish between stimuli with different current injections. The ability to discriminate sensations would open the door to a new stimulation paradigm where information can be encoded using stimulation waveform variation. Stimulation with non-rectangular waveforms remains an open challenge, thus to fully understand the potential impact of the waveform on neuroprosthetic interventions, further detailed experiments should be conducted.

## Data Availability

The simulated and measured data presented in this paper can be found at Open Science Framework (Collu, Earley, Barbaro, Ortiz-Catalan, 2022).
